# Novel target and cofactor repertoire for the transcriptional regulator JTY_0672 from *Mycobacterium bovis* BCG

**DOI:** 10.3389/fmicb.2024.1464444

**Published:** 2025-01-07

**Authors:** Hui Wang, Xiaotian Li, Shuxian Wang, Ren Fang, Jiayin Xing, Ruiying Wu, Chunhui Zhang, Zhaoli Li, Ningning Song

**Affiliations:** ^1^Weifang Key Laboratory of Respiratory Tract Pathogens and Drug Therapy, School of Life Sciences and Technology, Shandong Second Medical University, Weifang, China; ^2^SAFE Pharmaceutical Technology Co., Ltd., Beijing, China

**Keywords:** *Mycobacterium tuberculosis*, transcriptional regulation, JTY_0672, *JTY_3148*, cofactor

## Abstract

*Mycobacterium tuberculosis* (Mtb) is the pathogenic agent of tuberculosis (TB). Intracellular survival plays a central role in the pathogenesis of Mtb in a manner that is dependent on an array of transcriptional regulators for Mtb. However, the functionality of JTY_0672, a member of the TetR family of transcriptional regulators, remains unknown. In this study, EMSA, BIL, ChlP-PCR and animal models were used to investigate the regulation function of this protein. We found that the transcriptional regulator JTY_0672 is a broad-spectrum transcriptional regulatory protein and can directly regulate *JTY_3148*, both *in vitro* and *in vivo*. Cofactors containing V_*B*1_, V_*B*3_, V_*B*6_, V_*C*_, His, Cys, Asp, Glu, Fe^3+^, Pb^2+^, Cu^2+^, and Li^+^ were found to inhibit binding between JTY_0672 and the promoter of *JTY_3148*. JTY_0672 enhanced TAG production and increased Isoniazid (INH) resistance. Besides, this protein either promoted recalcitrance to the host immune response and induced pathological injury and inflammation. In summary, this research identified new targets and cofactors of JTY_0672 and deciphered the physiological functionality of JTY_0672. Our findings will provide an important theoretical basis for understanding the Mtb transcriptional regulatory mechanism.

## Introduction

Tuberculosis (TB) is caused by *Mycobacterium tuberculosis* (Mtb), a bacterium that mainly attacks the lungs through the respiratory tract. Approximately one-third of the global population is infected by Mtb, of these, 90–95% of infected individuals do not develop symptoms, while 5–10% of potentially infected individuals can develop active TB (Niu et al., 2024). The burden of treating TB is exacerbated by the spread of multidrug-resistant Mtb, there were approximately 160 000 deaths from multidrug/drug-resistant TB worldwide in 2022 bases on the World Health Organization (WHO) TB Report in 2023 (WHO, 2023).

To survive, Mtb have to face a range of stress factors such as temperature (Gauthier et al., 2021; Peruè et al., 2022; Madiraju et al., 2011), nutrients (Deb et al., 2009; Yousuf et al., 2015; Bharati et al., 2012), water (Mtetwa et al., 2022) and harmful substances (Alebouyeh et al., 2022; Varshney et al., 2024) and so on (Ramos et al., 2005). For instance, in the state of iron deficiency, transcriptomics revealed a stronger expression of genes involved to dormancy, which are enriched in key controls of the energy generation process (TCA cycle and cellular respiration) and ribosomal activity closure, thereby promoting Mtb dormancy. This suggests that environmental iron levels are a key stressor of bacterial transformation between *in vitro* proliferation and dormancy-like phenotypes (Alebouyeh et al., 2022). And wastewater may provide bacteria with nutrients needed for growth and lead to environmental transmission, direct exposure to the wastewater containing Mtb could potentially contribute to indirect transmissions which may lead to pulmonary or extra-pulmonary infections (Mtetwa et al., 2022). Besides, increased temperature caused decreased spleen and mesentery pathology in infected fish and decreased bacterial density in infected fish spleen. This indicate that mycobacterial infection may be limited by the heat tolerance of bacteria (Gauthier et al., 2021).

These stressful conditions can exert adverse effects on the growth of Mtb. Bacterial survival depends on rapid adaptive responses that are mediated by a series of regulatory proteins which provide appropriate regulation via physiological response (Ramos et al., 2005). In Mtb, there are a series of repair, tolerance and regulatory mechanisms to facilitate survival and compete with the host’s immune system. Of these mechanisms, transcription plays an important role for survival and pathogenicity under different conditions of stress (Galagan et al., 2013).

The tetracycline repressor (TetR) family of regulators (TRFRs) contain a C-terminal cofactor ligand structural domain and an N-terminal DNA-binding structural domain. TRFRs are involved in bacterial drug resistance, metabolism, antibiotic synthesis, and population sensing (Cuthbertson and Nodwell, 2013). Besides, the N-terminal arm unique to PhoP in Mtb plays an essential role in the expanded regulatory capabilities of this important regulator and is essential for phosphorylation-coupled transcription regulation of target genes (Das et al., 2013; Singh et al., 2023). For example, the knockdown of *Rv2160A* has been shown to inhibit intracellular redox reactions and promote the survival of Mtb in macrophages in an enriched lipid environment (García-Morales et al., 2022). BCG0878c, which directly regulates the expression of 3-methyladenine DNA glycosylase and influences the base excision activity of this glycosylase at the post-translational level (Liu et al., 2014). BCG_3893c (AotM) enhances the mycobacterial resistance against oxidative stress probably by inhibiting intracellular ROS production (Jiang et al., 2024). Besides, InbR, PrrA and DosR regulators in BCG are well studied (Yang et al., 2015; Mishra et al., 2017; Cui et al., 2022).

TRFRs usually bind to various ligands to sense environmental variation. Diverse ligand-binding capabilities increase the diversity of regulatory capabilities in TRFRs. Amino acids, vitamins, and metal ions, are all necessary for basic biological life activities. In *M. boivs*, CmtR functions as a novel redox sensor and that its expression can be significantly induced under H_2_O_2_ stress (Li et al., 2020). The binding of Rv3569c with Ser has shown to result in the reduced activity of enzymes involved in lipid metabolism in Mtb (Barelier et al., 2023). Rv1460 is a repressor of the *suf* operon in Mtb and plays an important role in the pathogenesis of iron homeostasis. △Rv1460 promotes the generation of biofilm and fails to grow under lower iron concentrations (Pandey et al., 2018). Vitamin C (V_*C*_) has been shown to inhibit Mtb growth by increasing the concentration of ferrous ion, thus resulting in DNA damage, reactive oxygen species (ROS) production, lipid changes and redox imbalance (Gaglani et al., 2023). It is suggested that V_*C*_ exerts impact on lipid synthesis and can reduce phospholipid content in Mtb to exert impact on Mtb survival (Syal et al., 2017).

JTY_0672 belongs to the TetR family and plays an important regulatory role in the life process of bacteria (Balhana et al., 2015). However, the regulatory function of JTY_0672 has yet to be elucidated. In this study, we found that the overexpression of JTY_0672 induced a significant up-regulation of *JTY_3148* (by 16-fold), as determined by qRT-PCR. *JTY_3148* (homologous with *rv3130c*, *tgs1*), acts as a diacylglycerol transferase that can accumulate triacylglycerol (TAG) which is known to be involved in Mtb dormancy (Sirakova et al., 2006). Mtb acquires fatty acids from the host for TAG synthesis, these fatty acids are subsequently stored as intracytoplasmic lipid inclusions (ILIs) to meet the carbon and nutrient requirements of bacteria during long-term persistence. Nitrogen deficiency can facilitate the induction of *rv3130c*-dependent TAG accumulation (Santucci et al., 2019). It has been reported that *rv3130c* is one of the most potent inducible genes by DosR during the non-replicative persistence of Mtb. The overexpression of *rv3130c* in *Mycobacterium maritimus* was also shown to enhance virulence in adult zebrafish (Liu et al., 2020). In addition, clinical experiments have shown that the expression of *rv3130c* is upregulated and the lipid content of macrophages is increased in TB patients treated with Fenofibrate, thus suggesting that this treatment promotes intracellular Mtb persistence and places patients at a higher risk of death (Santucci et al., 2019). In addition, *rv3130c* is known to be involved in drug resistance and is upregulated in MDR in response to isoniazid, thus suggesting that this gene could represent a new candidate for future drug-resistant TB monitoring and treatment (Yimcharoen et al., 2022).

In the present study, we found that JTY_0672 can regulate the expression of multiple genes, including *JTY_3148*, both *in vitro* and *in vivo*. C13 base is the key base by which JTY_0672 interacts with the *JTY_3148* promoter. Furthermore, a range of cofactors can all affect the binding ability of JTY_0672 with the *JTY_3148* promoter. Furthermore, JTY_0672 promotes the growth and colonization of MS in animal organs, promotes inflammatory responses, and causes pathological damage, thus suggesting that JTY_0672 plays an important role in pathogenicity. Collectively, this study enhance our understanding of the transcriptional regulation role of JTY_0672.

## Materials and methods

### Animals, bacterial strains, and plasmids

C57BL/6 mice were purchased from Jinan Pengyue Laboratory Animal Breeding Ltd (Jinan, China). *Escherichia coli* BL21 (λDE3) was grown in flasks using LB broth. *Mycobacterium bovis* BCG Tokyo 172 (BCG) and MS strains were grown in 7H9 broth containing 0.05% Tween 80 and 10% Oleic-Albumin-Dextrose-Catalase (OADC). The recombinant plasmid pET22b-JTY_0672 was used to express protein while pMV262-JTY_0672-MS and pMV262-JTY_0672-BCG were used for overexpression studies. Both pMV262-JTY_0672-BCG and pMV262-JTY_0672-MS strains were overexpressed by heating at 45°C for 2 h.

### Ethics statement

Wild-type (WT) C57BL/6 mice (6 weeks) were purchased from Jinan Pengyue Laboratory Animal Breeding Ltd (Jinan, China). All mice were housed in a specific pathogen-free (SPF) environment in accordance with standard humane animal husbandry protocols which were approved by the Ethical Committee of Animal Experiments of Shandong Second Medical University (Ethical Committee Approval number 2022SDL109).

### Expression and purification of recombinant protein

The recombinant pET22b-JTY_0672 plasmid was transformed in to BL21 (λDE3) cells and cultured in LB broth medium at 37°C at 180 rpm. Isopropyl-β-D-thiogalactopyranoside (IPTG) was added at a final concentration of 1 mM when the OD_600_ reached 0.7 to induce gene expression for 12 h at 30°C. Subsequently, bacteria were harvested by centrifugation, resuspended with lysis buffer by the addition of lysozyme, sonicated in ice, and then centrifuged at 10,000 *g* for 30 min. The supernatant was then passed through the affinity column with 6 Fast Flow Ni-NTA (Solarbio, Beijing, China) and washed by wash buffer containing gradient imidazole and elution buffer. Protein concentrations were determined by the BCA protein assay (TIANGEN, Beijing, China).

### Electrophoretic mobility shift assays

Electrophoretic mobility shift assays (EMSA) were performed to confirm the interaction between JTY_0672 and targeted genes from BCG. We synthesized Cy5-labeled probes (Supplementary Table 1), and targeted gene promoters were amplified by PCR using BCG genomic DNA as a template. JTY_0672 and PCR fragments from targeted genes were incubated in binding buffer (20 mM Tris-HCl, 150 mM NaCl, 1 mM DTT, and 5% glycerol) at 37°C for 20 min. After incubation, the complex was loaded and analyzed on 8% non-denatured polyacrylamide gels in 1 × Tris-Borate-EDTA buffer at 200 V for 4 h. For cofactor tests, we added ligands at various concentration gradients in binding buffer. All EMSA images were acquired by Bio-OI Scan software (Guangyi, Guangzhou, China).

### cDNA synthesis and quantitative real-time polymerase chain reaction

The primers used for quantitative polymerase chain reaction (qPCR) are shown in Supplementary Table 1. RNA isolation was performed as described previously. Briefly, the frozen cell pellets were suspended in 1 mL of TRIzol reagent (Life Technology), and the cells were disrupted by sonication for 3 min on ice. This mixture was centrifuged at maximal speed for 1 min, and the supernatant was transferred to a 2-mL Heavy Phase Lock Gel tube (TIANGEN) containing 300 mL chloroform–isoamyl alcohol (24:1), inverted rapidly for 15 s, incubated for 2 min, and centrifuged at maximal speed for 5 min. The RNA in the aqueous phase was then precipitated with 270 mL isopropanol and 270 mL of a high-salt solution (0.8 M Na citrate and 1.2 M NaCl) at 4°C overnight. RNA was purified using an RNeasy Mini Kit following the manufacturer’s recommendation (QIAGEN) with one on column DNase I treatment (QIAGEN) at 37°C for 30 min to remove any contaminating gDNA. DNase I was removed with an RNeasy Mini Kit according to the cleanup procedure.

First-strand cDNA was synthesized from 20 ng RNA with SuperScript III First-Strand Synthesis SuperMix kit (Invitrogen), according to the manufacturer’s instructions. Quantitative realtime PCR (qRT-PCR) was carried out in a LightCycler480 II (Roche) RT–PCR machine with an iQTM SYBR Green Supermix (Bio-Rad) according to previous study (Song et al., 2016). The PCR conditions were as follows: 95°C for 3 min, 40 cycles at 95°C for 10 s, 55°C for 30 s, 72°C for 10 s, and one cycle at 50°C for 3 min. Reactions were performed with 12.5 μL SYBR Green Supermix and 20-fold diluted cDNA in a total volume of 25 μL. All the qRT-PCR assays were carried out in technical duplicates for three biological replicates with a no-template and a no-RT control. *JTY_2708*, encoding *sigA*, was used as a housekeeping gene for normalization. The efficiencies of gene-specific primer pairs were calculated by measuring the slope of a linear regression curve that resulted from Cq values for a 10-fold dilution series with gDNA as the template. The enriched and input DNA samples from ChIP were analyzed by PCR and quantitative polymerase chain reaction (qPCR) using the primers shown in Supplementary Table 1. The enriched promoter of *JTY_3148* s was normalized to 16S ribosomal RNA (rRNA). The relative expression folds were calculated using the 2^–△^^△^ *^Ct^* method (Cui et al., 2022).

### Bio-layer interferometry

Biotin-labeled primers (Bio-SN494F/Bio-SN494R) (Supplementary Table 1) were used to amplify the *JTY_3148* promoter DNA fragment for BLI. The biotin-labeled DNA fragments were fixed onto streptavidin (SA) sensor probes (Sartorius, Shanghai, China) and soaked in PBST for at least 10 min. JTY_0672 was diluted to 312.5–10 000 nM in duplicate. The initial baseline was acquired by incubating the biosensor with PBST buffer for 60 s. Then, 60 nM of DNA fragments tagged with biotin was loaded onto the SA biosensors for 60 s. After loading, the biosensor was transferred back to the buffer used for baselining for 60 s. Then, to evaluate the association between DNA and protein, the SA biosensors were placed into a gradient protein buffer for binding for 90 s. Finally, complex dissociation was monitored by transferring the biosensor back into PBST for 90 s. Data Analysis version 12.0 software was used to calculate the KD value.

### Chromatin immunoprecipitation assays

Chromatin immunoprecipitation (ChIP)–PCR was performed in accordance with a method described previously (Cui et al., 2022). In brief, BCG cultures were allowed to reach to logarithmic growth phase and were then fixed with 1% formaldehyde for 10 min. Then, the reaction was quenched by the addition of glycine at a final concentration of 125 mM. Cross-linked cells were resuspended with IP buffer and sonicated for 5 min at 20% amplitude with an ultrasonic crusher. Next, the anti-JTY_0672 polyclonal antibody was conjugated with M-280 Sheep anti-rabbit beads (Thermo Scientific, Waltham, MA) overnight at 4°C. All samples were purified with an iPure DNA extraction kit (Diagenode, Besancon, France) in accordance with the manufacturer’s instructions. Purified DNAs were then used as templates for PCR amplification.

### Binding site prediction

Based on the JTY_0672 binding motifs predicted previously by pMV262-JTY_0672-BCG and pMV262-BCG transcriptome analysis (data not shown) and the positive candidates showing binding in EMSA, we designed and constructed a new sequence logo using MEME.^1^ Subsequently, PSPM data were submitted to MAST^2^ to search for extra pseudo-JTY_0672 binding sites with regards to published bacterial one-hybrid (BIH) data (Zeng et al., 2012).

### Model construction and molecular docking

In order to screen the key amino acid residues of JTY_0672 that interact with cofactors, we next determined the three-dimensional (3D) pattern diagram of JTY_0672 and used molecular docking to investigate the mode of binding with cofactors. The 3D structure of JTY_0672 was simulated by the Alphafold2^1,2^ model, and the 3D structure, the cofactors were obtained from the PUBCHEM database, and energy was minimized under the MMFF94 force field. Proteins were pre-treated using PyMol 2.5.2^4^ to remove water, salt ions, and cofactors. Then, we set up the docking box to wrap around the entire protein structure. When docking, the global search had a level of detail of 32, while other parameters remain default. On this basis, PyMol2.5.2^4^ was used for computer simulation and visual analysis.

### Growth detection

To investigate the effect of JTY_0672 on growth, the pMV262-MS and pMV262-JTY_0672-MS strains were inoculated in 7H9 broth containing kanamycin (50 μg/mL) to an OD_600_ value of 0.5 and then heated at 45°C for 2 h. Then, the cells were placed at 37°C for 144 h with shaking. The culture was monitored at various time points (every 12 h) and a 100 μL sample was taken to determine the CFU on 7H10 agar plates at each time point, this allowed us to generate a growth curve. The different cofactors (V_*C*_, Fe^3+^, Cu^2+^) were added as indicated to test the cofactor effect on pMV262-JTY_0672-MS strain growth.

### IC50 detection

IC50 was determined by broth microdilution assay as previously described with minor modification (Ouyang et al., 2024). The exponential-phase cell cultures (OD_600_ = 0.5) of pMV262-MS and pMV262-JTY_0672-MS were diluted 10 times, which were afterward heated at 45°C for 2 h and then incubated at 37°C with shaking. Next, the samples were diluted 1:10 into 7H9 medium, and then distributed in a 96-wells plate. Isoniazid (INH) was tested at 62.5 μg/mL in the first row, then serially diluted with a factor 2. OD_600_ of cultures were measured by spectrophotometer after a 48-h-incubation at 37°C with shaking. IC50 were calculated using GraphPad Prism. Experiments were performed with biological triplicates.

### Mouse infection and colony-forming unit counting

The C57BL/6 mice were challenged with pMV262-JTY_0672-MS by tail-vein injection. The control group of mice were challenged with pMV262-MS or PBST (PBS with 0.05% Tween 80). Each mouse was injected with 5 × 10^6^ CFU. At 4 d, 7 d, 14 d, and 21 d post-infection, we obtained lung, liver, spleen and kidney tissue from each mouse in a sterile manner. Then, each sample was homogenized in PBS buffer. Next, the homogenates were serially diluted and plated on Middlebrook 7H10 agar plates. Bacterial burden was then determined by determining CFU after incubation for 3 days at 37°C.

### Pathology and histopathological analyses

Tissue samples of lung, liver, spleen and kidney were obtained from each mouse and fixed in 4% PFA for 24 h. Then, the tissues were dehydrated, embedded in paraffin, and sectioned. Sections were then mounted on slides and stained with hematoxylin and eosin (H&E) to permit pathological analysis.

### Quantification of inflammatory factor levels

Based on previous results of organ colonization, on day 4 and 7 post-infection, we determined the levels serum cytokines (IL-1β, IL-6, IFN-γ and TNF-α) with Mouse ELISA Kits (Solarbio, Beijing, China) in accordance with the manufacturer’s instructions. Eye blood was harvested from infected mice and serum samples were collected via centrifugation at 1,000 *g* for 20 min. The absorbance at 450 nm was then determined using a microplate spectrophotometer.

### Statistical analysis

Experimental data were analyzed by *t*-test and one-way analysis of variance (ANOVA) in GraphPad Prism 8 software. A *p*-value of < 0.05 was considered to be indicative of a statistically significant result. ns stands for not significant, *p* > 0.05; *p < 0.05; **p < 0.01; ***p < 0.001; ****p < 0.0001.

## Results

### JTY_0672 is a broad-spectrum regulatory protein

**FIGURE 1:**
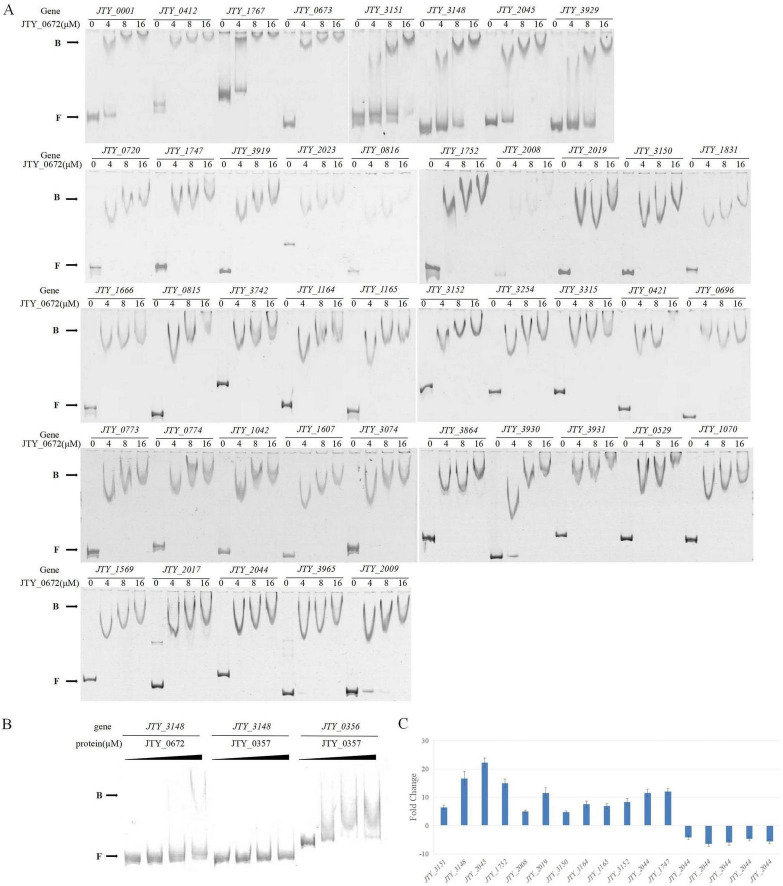
**(A)** The binding of JTY_0672 with target gene promoters, as determined by EMSA, with indication of JTY_0672 concentrations (in μM). **B** stands for JTY_0672–DNA complexes, **F** stands for free DNA. **(B)** EMSA assays confirming the binding specificity between JTY_0672 and the *JTY_3148* promoter. Protein JTY_0357 was used as a negative control for *JTY_3148* promoter. Protein JTY_0357 binds with *JTY_0356* promoter. **(C)** Relative expression levels of target genes, as detected by qRT-PCR. Relative expression level is given as fold change of the expression level in pMV262-JTY_0672-BCG overexpression strain versus the expression level of pMV262-BCG wild strain.

Based on protein function and fold change of genes in the pMV262-JTY_0672-BCG and pMV262-BCG primary transcriptomic analysis (Table 1 and Supplementary Table 2), we selected 43 genes for the EMSA test (Supplementary Table 3). As shown in Figure 1A, the protein JTY_0672 has different binding abilities to different promoters, thus indicating that JTY_0672 is a broad-spectrum regulatory protein, as we hypothesized. Additionally, the protein JTY_0357 and the promoter of *JTY_0356* were used as controls for binding specificity confirmation (Figure 1B).

**TABLE 1:** The function and fold change of genes in pMV262-JTY_0672-BCG overexpression strain by transcription analysis.

Gene	Function	log2 fold change
*JTY_0672*	Transcriptional regulator	5.6416
*JTY_0001*	Chromosomal replication initiator protein DnaA	−0.027505
*JTY_0412*	Long-chain-fatty-acid–AMP ligase FadD30	0.68777
*JTY_1767*	PPE family protein PPE24	0.44927
*JTY_0673*	Carotenoid cleavage oxygenase	−1.1816
*JTY_3151*	Two component transcriptional regulator DevR (DosR)	1.5956
*JTY_3148*	Diacylglycerol O-acyltransferase	3.4064
*JTY_2045*	Alpha-crystallin HspX	4.0777
*JTY_3929*	ESX-1 secretion-associated protein EspE	−1.5877
*JTY_0720*	30S ribosomal protein S17	−0.42136
*JTY_1747*	Transmembrane protein	3.0037
*JTY_3919*	Monooxygenase EthA	−1.5871
*JTY_2023*	Hypothetical protein	−1.2117
*JTY_0816*	Monooxygenase	−1.1253
*JTY_1752*	Hypothetical protein	3.7301
*JTY_2008*	Universal stress protein	1.6244
*JTY_2019*	Ferredoxin FdxA	3.1965
*JTY_3150*	Two component sensor histidine kinase DevS	1.5792
*JTY_1831*	Hypothetical protein	2.1637
*JTY_1666*	N-acetyl-gamma-glutamyl-phosphate reductase argC	−0.99675
*JTY_0815*	Transcriptional regulator	−0.93928
*JTY_3742*	Bifunctional penicillin-insensitive transglycosylase/penicillin-sensitive transpeptidase ponA2	−0.43877
*JTY_1164*	2-methylcitrate dehydratase PrpD	2.722
*JTY_1165*	Methylcitrate synthase PrpC	3.1164
*JTY_3152*	Universal stress protein	2.2968
*JTY_3254*	Stearoyl-CoA 9-desaturase desA3	1.7857
*JTY_3315*	L-lysine-epsilon aminotransferase	1.198
*JTY_0421*	Membrane protein	−0.85154
*JTY_0696*	Membrane protein MmpS5	−1.2257
*JTY_0773*	Acyl-CoA dehydrogenase FadE9	−1.2333
*JTY_0774*	Methylmalonate-semialdehyde dehydrogenase	−1.4018
*JTY_1042*	Polyketide synthase	−1.7582
*JTY_1607*	Quinolinate synthetase A nadA	−1.0457
*JTY_3074*	Hypothetical protein	−1.9595
*JTY_3864*	Polyketide synthase	−1.1537
*JTY_3930*	ESX-1 secretion-associated protein EspF	−1.4176
*JTY_3931*	ESX-1 secretion-associated protein EspG	−1.3835
*JTY_0529*	Anti-anti-sigma factor	1.7741
*JTY_1070*	PE family protein PE8	1.0727
*JTY_1569*	Hemoglobin GlbN	2.1507
*JTY_2017*	Universal stress protein	1.6107
*JTY_2044*	Hypothetical protein	3.1609
*JTY_3865*	Long-chain-fatty-acid–AMP ligase FadD32	−1.2288
*JTY_2009*	Cation transporter ATPase F ctpF	1.3145

Based on the results of EMSA validation, 17 potential significant target genes regulated by JTY_0672 were identified for qRT-PCR analysis. Of these 17 targets, up-regulation occurred in 12 genes (*JTY_3151*, *JTY_3148*, *JTY_2045*, *JTY_1752*, *JTY_2008*, *JTY_2019*, *JTY_3150*, *JTY_1164*, *JTY_11645*, *JTY_3152*, *JTY_2044*, *JTY_1747*), while down-regulation occurred in five genes (*JTY_0673*, *JTY_3929*, *JTY_3919*, *JTY_0696*, *JTY_3931*) (Figure 1C). These results demonstrated that JTY_0672 can act as an activator as well as a repressor.

### JTY_0672 bound to the *JTY_3148* promoter both *in vitro* and *in vivo*

**FIGURE 2:**
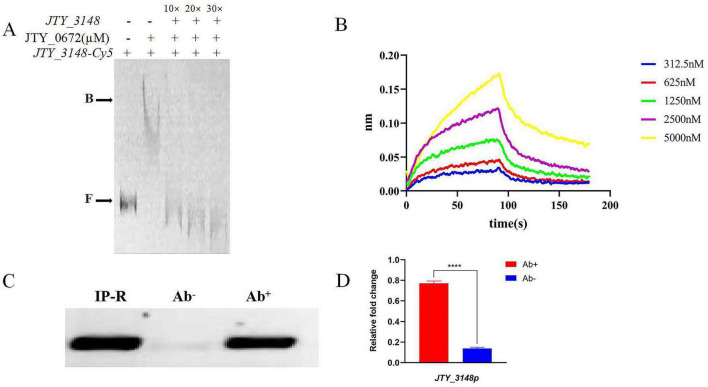
Specific binding of JTY_0672 to the *JTY_3148* promoter *in vitro* and *in vivo*. **(A)** The addition of ten to thirty-fold excess cold probes allowed Cy-5 labeled *JTY_3148* to be out-competed due to its inability to bind the protein. **B** stands for JTY_0672–DNA complexes, **F** stands for free DNA. The protein concentration of JTY_0672 is 16 μM. **(B)** BLI detection of the interaction between JTY_0672 and the *JTY_3148* promoter. The *x*-axis represents time, unit in seconds, and the *y*-axis represents the signal value for protein and DNA binding. **(C)** ChIP-PCR verifying that JTY_0672 bound to the *JTY_3148* promoter *in vivo*. ChIP using a specific antibody (Ab+) or pre-immune antibody (Ab–) to precipitate the immuno-complex. Lane 1, IP-R input; Lane 2, pre-immune antibody (Ab–); Lane 3, specific antibody (Ab+) for JTY_0672. **(D)** Relative expression levels of *JTY_3148* promoter, as detected by qRT-PCR. Ab+ stands for samples treated with antibody; Ab– stands for control samples. ****p < 0.0001.

The specific binding of JTY_0672 to the *JTY_3148* promoter was verified by competitive inhibition experiments. As shown in Figure 2A, a series of unlabeled cold probes was added to the reaction as competitors. The excess of cold probes bound to JTY_0672, thus resulting in the reduction of complex formation. These results demonstrated that JTY_0672 could bind to the *JTY_3148* promoter *in vitro*.

To further confirm the binding of JTY_0672 to the *JTY_3148* promoter *in vitro*, we used biotin-labeled *JTY_3148* promoter DNA to intercalate with gradient-diluted JTY_0672. BLI showed that the dissociation constant (KD) was 2.199 × 10^–6^ M (Figure 2B), thus demonstrating that JTY_0672 binds with the *JTY_3148* promoter *in vitro*.

Next, we used ChIP-PCR and ChIP-qPCR to investigate whether JTY_0672 binds to the *JTY_3148* promoter *in vivo*. As shown in Figure 2C, PCR product size of the *JTY_3148* promoter were detected in both the IP-R input and the experimental group with the addition of specific antibody to JTY_0672. No bands were evident in the control group (which was devoid of antibody). ChIP-qPCR either demonstrated that change folds is higher in antibody added sample (Ab+) than control (Ab−) in Figure 2D. Overall, these data indicated that JTY_0672 bound to the *JTY_3148* promoter *in vivo*.

### C13 was an important base for the interaction of JTY_0672 with the *JTY_3148* promoter

**FIGURE 3:**
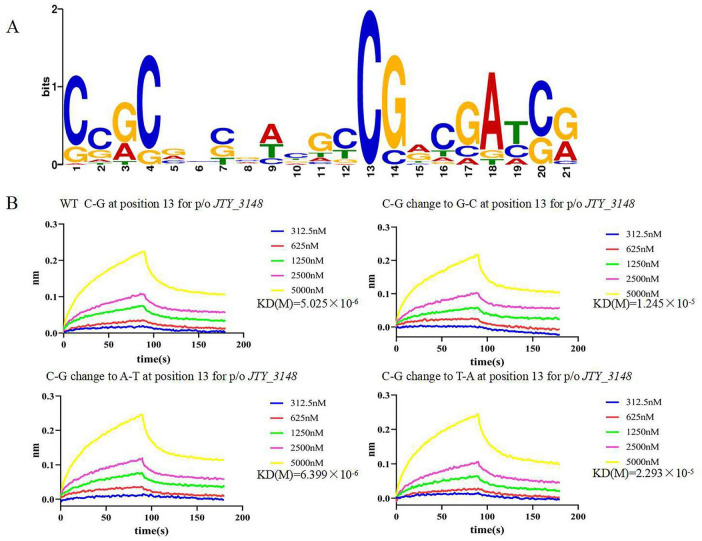
Systematic base pair (bp) substitution in the JTY_0672 consensus sequence to analyze the sequence specificity of binding. **(A)** Graphical representation of the sequence logo. The height of the stack of letters corresponds to the information content (bits). The *x*-axis represents the position of the nucleotide, and the *y*-axis represents the conservation of the base at that position. **(B)** BLIs of JTY_0672 binding to variants harboring a single-bp substitution at position 13. The bp change is given at the top of each gel along with JTY_0672 concentrations (in nM). The *x*-axis represents time, unit in seconds, and the *y*-axis represents the signal value for protein and DNA binding. KD values are indicated on the right of the figures.

EMSA showed that JTY_0672 can interact with the promoter of various target genes *in vitro*. MEME software was used to construct a conserved sequence logo which was 21 bp in length (Figure 3A); the horizontal coordinate represents the position of the base, and the vertical coordinate represents the conservation of the base at that position. Since position C13 was highly conserved; we used this position for further testing. As shown in Figure 3B, the mutation of C13 in the *JTY_3148* conserved binding box exhibited a different binding affinity with JTY_0672. WT C-G at position 13 for *JTY_3148* showed binding affinity for JTY_0672 (KD = 5.025 × 10^–6^ M). In comparison, when the C13 position of *JTY_3148* was mutated to G13, A13 and T13 showed lower binding affinity (KD = 1.245 × 10^–5^ M, 6.399 × 10^–6^ and 2.293 × 10^–5^ M, respectively). These data demonstrated that C13 plays a key role in the interaction of JTY_0672 with the *JTY_3148* promoter.

### Cofactor specificity of JTY_0672

**FIGURE 4:**
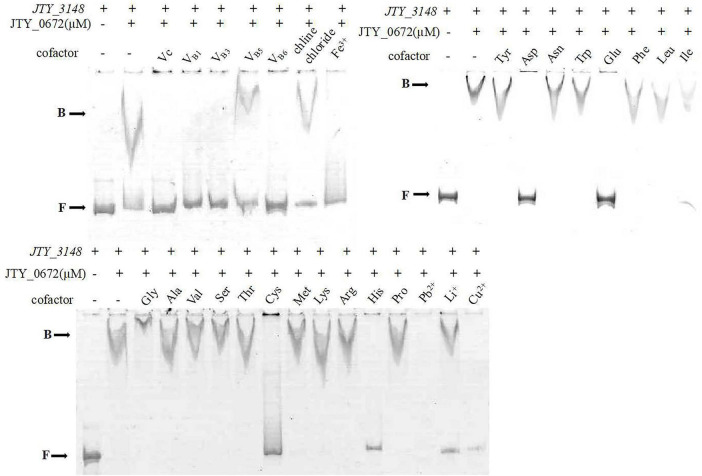
The effects of different cofactors on the binding of JTY_0672 to the *JTY_3148* promoter, as determined by EMSA. The positions of free DNA (**F**) and JTY_0672–DNA complexes (**B**) are highlighted. The promoter of *JTY_3148* was added in each well of the EMSA Gels. – stands for no protein was added. + stands for protein was added with the same concentration of JTY_0672 (in μM). In the top row above the gels, cofactors are displayed (V_C_, V_B6_, Cys, Cu^2+^ with 10 mM, Fe^3+^ with 0.4 mM, the other cofactors had a final concentration of 80 mM).

**FIGURE 5:**
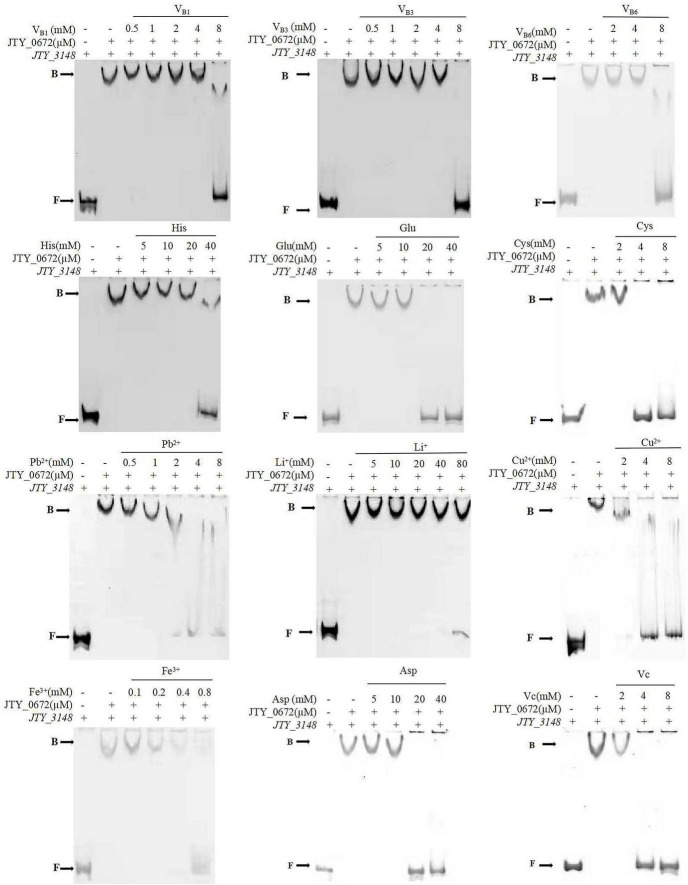
The effect of different concentration gradients of a selection of cofactors on the binding of JTY_0672 to the *JTY_3148* control region by EMSA. The positions of free DNA (**F**) and JTY_0672–DNA complexes (**B**) are highlighted. The promoter of *JTY_3148* was added in each well of the EMSA Gels. – stands for no protein was added. + stands for protein was added with the same concentration of JTY_0672 (in μM). All cofactor concentrations are displayed in mM.

Transcriptional regulators usually bind with cofactors to sense environmental changes to regulate gene expression. In this study, a series of cofactors containing amino acids, vitamins, and metal ions, were used to test their relative effects on the binding of JTY_0672 to the *JTY_3148* promoter by EMSA. The ESMA results showed that a range of cofactors (V_*B*1_, V_*B*3_, V_*B*6_, V_*C*_, His, Cys, Asp, Glu, Fe^3+^, Pb^2+^, Cu^2+^, and Li^+^) could inhibit the binding of JTY_0672 to the *JTY_3148* promoter (Figure 4). Based on preliminary results in Figure 4, we used a gradient of cofactor concentrations to further test binding affinity. EMSA results showed that Li^+^ influenced the binding of JTY_0672 with the *JTY_3148* promoter at 80 mM, His at 40 mM, Glu and Asp at 20 mM, V_*B*1_, V_*B*3_ and V_*B*6_ at 8 mM, Cys, V_*C*,_ Cu^2+^, and Pb^2+^ at 4 mM, Fe^3+^ at 0.8 mM, respectively (Figure 5).

### Molecular docking

**FIGURE 6:**
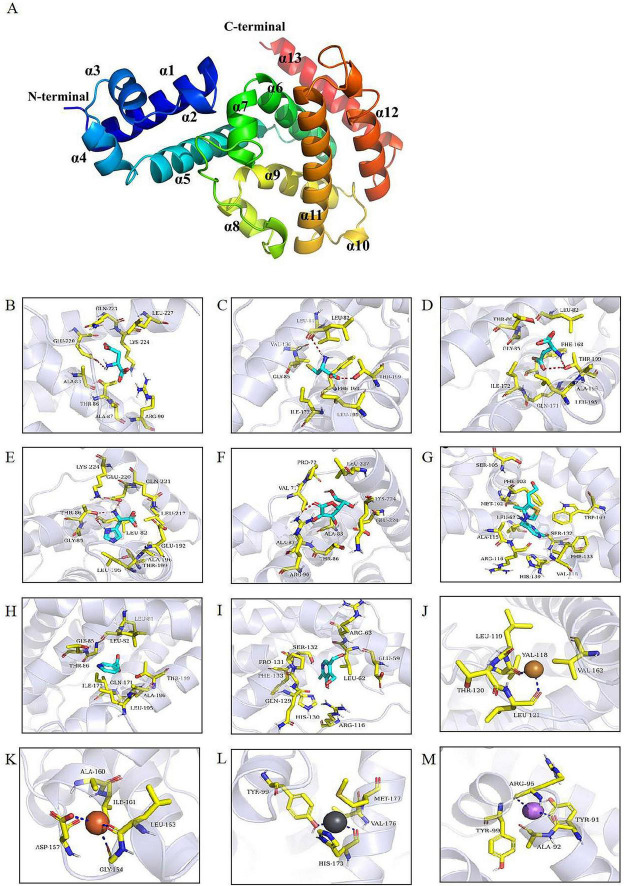
**(A)** The 3D structural model of JTY_0672. α1-α13 helices are shown in different colors. The N and C terminals are marked. **(B–M)** Molecular docking identified key amino acid residues. The interaction of JTY_0672 with **(B)** Asp, **(C)** Cys, **(D)** Glu, **(E)** His, **(F)** V_B1_, **(G)** V_B3_, **(H)** V_B6_, **(I)** V_C_, **(J)** Cu^2+^, **(K)** Fe^3+^, **(L)** Li^+^, **(M)** Pb^2+^. Asp, Cys, Glu, His, Vc, V_B1_, V_B3_, V_B6_ are shown as cyan, Cu^2+^ is shown as yellow, Fe^3+^ is shown as brown, Pb^2+^ is shown as bluish gray, Li^+^ is shown as violet, the structure of residues near the cofactor is shown as yellowish, and the backbones of the receptor proteins are shown as transparent blue bands. The red dashed line indicates hydrogen bonding and the dark blue dashed line indicates metal complexation.

The 3D structure of JTY_0672 revealed that the JTY_0672 possess 13 α-helices, specific structure and interval analyses are shown in Figure 6A and Table 2.

**TABLE 2:** JTY_0672 protein interval analysis.

Structure	Amino acid residue intervals	Structure	Amino acid residue intervals
α*1*	Q4-H22	α*8*	V118-T120
α*2*	P24-L27	α*9*	P131-L145
α*3*	T29-A36	α*10*	T151-L153
α*4*	T40-F47	α*11*	D158-L180
α*5*	M50-A68	α*12*	L195-A205
α*6*	P77-E94	α*13*	P210-R229
α*7*	R95-M102		

As shown in Figures 6B–M, molecular docking predicted the binding pattern of cofactor ligands to JTY_0672 protein. His and V_*B*3_ interacted with residue Leu-82, Asp and V_*C*_ interacted with residue Arg-90, Cys and Glu interacted with residue Thr-199, and His and Asp interacted with residue Glu-220. These results indicate that Leu-82, Arg-90, Thr-199, and Glu-220 are likely to be the key potential amino acid residues that allow JTY_0672 to interact with cofactors.

Table 3 shows the scores for the binding of JTY_0672 to cofactors, as predicted, in which a negative binding energy of cofactors and proteins indicates the possibility of binding, and a lower value binding energy indicates a better binding effect. It is evident that the binding energies of all substances are negative, thus suggesting that cofactors and proteins were able to bind. In comparison, the binding effect of cofactors was more effective, especially V_*B*1_, V_*B*3_, V_*B*6_, V_*C*_, for which the binding energy value was less than -5 kcal/mol, thus indicating that the binding ability with JTY_0672 was stronger.

**TABLE 3:** The docking score of JTY_0672 with cofactors.

Ligands	Docking_ score (kcal/mol)	Ligands	Docking_ score (kcal/mol)
Asp	−4.6	Cu^2+^	−1.3
Cys	−3.9	Fe^3+^	−1.4
Glu	−5.2	V_B1_	−5.4
His	−5.1	V_B3_	−5.3
Li^+^	−1.1	V_B6_	−5.3
Pb^2+^	−1.2	V_C_	−5.4

### JTY_0672 promote TAG concentration and isoniazid IC50

*JTY_3148* (homologous with *rv3130c*, *tgs1*), acts as a diacylglycerol transferase that can accumulate triacylglycerol (TAG) which is known to be involved in Mtb dormancy, therefore, we tested the TAG concentration in pMV262-JTY_0672-MS and pMV262-MS strains. Overall, the TAG concentration of pMV262-JTY_0672-MS was higher than pMV262-MS. Especially at 48 h, the TAG concentration in pMV262-JTY_0672-MS was significantly higher than pMV262-MS, and the concentration of pMV262-JTY_0672-MS and pMV262-MS is 0.51 mg/10^8^ cell and 0.28 mg/10^8^ cell, respectively (Supplementary Figure 1).

Besides, *rv3130c* is known to be involved in drug resistance and is upregulated in MDR in response to isoniazid, therefore, we calculated the isoniazid IC50 to compare the values between pMV262-MS and pMV262-JTY_0672-MS. And the results show that IC50 of pMV262-MS and pMV262-JTY_0672-MS is 5.812 and 9.144 μg/mL, respectively (Supplementary Figure 2).

### JTY_0672 promoted recalcitrance to the host immune response

**FIGURE 7:**
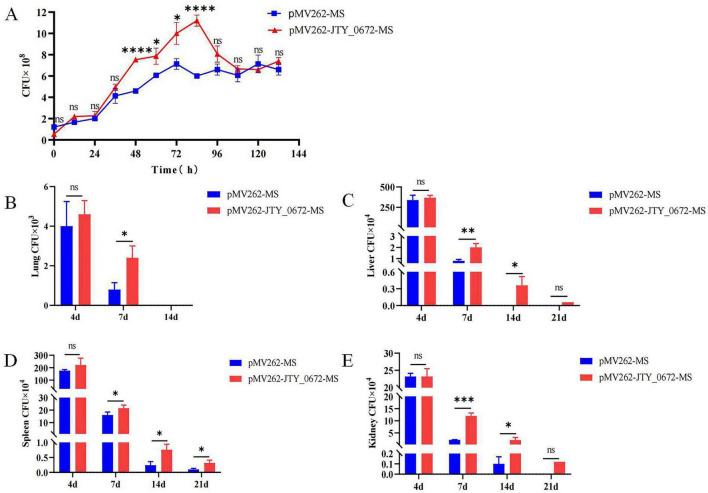
**(A)** Growth curves for the pMV262-JTY_0672-MS and pMV262-MS strains. The growth curve was created using times as the *x*-axis and the number of bacteria as the *y*-axis. Each sample was analyzed in triplicates. Error bars represent mean ± SEMs (*n* = 3), ns stands for not significant, *p* > 0.05; *p < 0.05; **p < 0.01; ***p < 0.001; ****p < 0.0001. **(B–E)** Colonization of pMV262-JTY_0672-MS overexpression strains in mouse organs. **(B)** Lung. **(C)** Liver. **(D)** Spleen. **(E)** Kidney. Error bars represent means ± SEMs (*n* = 6). The *x*-axis represents time, and the *y*-axis represents the number of bacteria.

To determine if JTY_0672 can influence the growth of MS, we next used pMV262-JTY_0672-MS overexpression strains to investigate growth curves. According to the bacterial growth curve, the first 24 h belong to the adaptation period. When the microorganisms are inoculated to the fresh culture medium, the number of cells has not increased to a certain extent during the beginning of culture, due to the need of the metabolic system to adapt to the new environment, so it shows an adaptation period. After 24 h, with the growth of the bacteria, the concentration of the protein JTY_0672 in pMV262- JTY_0672-MS strain increased, resulting in expression changes of targeted genes regulated by JTY_0672, causing growth rate accelerate. After 100 h, the bacteria enter stationary period, due to the concentration variation of JTY_0672 protein, the expression level of targeted genes regulated by JTY_0672 protein either changed, causing complex regulatory systems to reduce growth rate. The results show that significant differences occurred between pMV262-MS and pMV262-JTY_0672-MS from 48 to 84 h, thus indicating that the overexpression of JTY_0672 induced the growth of MS (Figure 7A). In addition, we either tested the effect of cofactors such as Vc, Fe^3+^ and Cu^2+^ for pMV262-JTY_0672-MS strain growth. The results show that cofactors such as V_*C*_, Fe^3+^, Cu^2+^ can inhibit the pMV262-JTY_0672-MS overexpressing strain growth *in vitro* (Supplementary Figure 3).

To investigate whether JTY_0672 can influence colonization in the host, we next used a mouse model of Mtb infection. We injected C57BL/6 mice with 5 × 10^6^ CFUs of MS per mouse via tail vein injection. The data imply that JTY_0672 offers no advantage on colonization of bacteria to organs. However, the data suggest after Day 4, the host responds to clear the infection, pMV262-JTY_0672-MS has better survival compared to empty vector. Therefore, JTY_0672 offer recalcitrance to the host immune response (Figures 7B–E). These results revealed that JTY_0672 activated the inflammatory response more strongly than the empty vector or PBST control.

### JTY_0672 inhibited the inflammatory response and induced organ injury in mice

**FIGURE 8:**
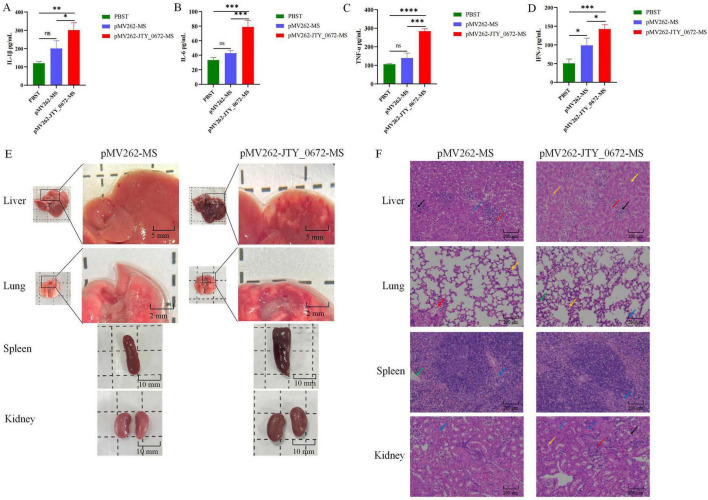
**(A–D)** Cytokine levels in the serum of infected 7 day mice. **(A)** IL-1β. **(B)** IL-6. **(C)** TNF-α. **(D)** IFN-γ. The detection of cytokine levels from the immunized mice were performed using ELISA. Each sample was analyzed in triplicates. Error bars represent means ± SEMs (*n* = 3). Time is shown as the *x*-axis, and the number of cytokine levels as the *y*-axis. ns stands for not significant, *p* > 0.05; *p < 0.05; **p < 0.01; ***p < 0.001; ****p < 0.0001. **(E)** Histological lesions in the liver, lungs, spleen and kidneys of mice infected with pMV262-JTY_0672-MS and pMV262-MS. Scale bars are 2 mm, 5 mm and 10 mm. **(F)** Histopathological sections taken from the liver, lungs, spleen and kidneys in mice infected with pMV262-JTY_0672-MS and pMV262-MS strains. In liver, black arrows indicate cell necrosis, red arrows indicate cell infiltration, and yellow arrows indicate cell steatosis. In lung, blue arrows indicate cellular infiltration, purple arrows indicate eosinophils, orange arrows indicate macrophage exudation, and green arrows indicate leukocytes. In spleen, blue arrows indicate loose cell arrangement, green arrows indicate sinusoid expansion. In kidney, blue arrows indicate cell water pattern degeneration, orange arrows indicate eosinophilia, purple arrows indicate basophilic cell aggregation, and red arrows indicate cell infiltration. The scale bars are 200 μm.

In order to investigate the role of JTY_0672 in the inflammatory response, we considered the colonization data from organs, and measured the levels of IL-1β, IL-6, TNF-α, and INF-γ in the sera of mice on day 7 after infection. As shown in Figures 8A–D, the levels of the proinflammatory cytokines IL-1β, IL-6, TNF-α, and IFN-γ were significantly higher (*p* < 0.05) than those observed in the control group of mice (only injected with PBST or MS). These results revealed that JTY_0672 activated the inflammatory response. The levels of IL-1β, IL-6, TNF-α, and INF-γ in the sera of mice on day 4 after infection were either tested and the results are shown in Supplementary Figure 4.

Next, we investigated tissue and pathological sections from mice after infection. Analysis showed that the area of hemorrhage in the lungs of pMV262-JTY_0672-MS-infected mice was larger than that of pMV262-MS. The livers of both pMV262-JTY_0672-MS and pMV262-MS mice showed hemorrhagic spots, the livers of pMV262-JTY_0672-MS appeared to contain a wide distribution of white granulomas of various sizes. Enlargement of the spleen occurred in both pMV262-JTY_0672-MS and pMV262-MS mice, although enlargement was more notable in the pMV262-JTY_0672-MS mice. There was no difference in kidney size when compared between pMV262-JTY_0672-MS and pMV262-MS mice (Figure 8E).

Pathological liver sections revealed that pMV262-JTY_0672-MS mice possessed multifocal necrosis of a large number of hepatocytes, cytosolic solidification and lysis of the nucleus, unclear cell demarcation, irregular arrangement, a small number of infiltrating lymphocytes and granulocytes, and a small number of hepatocytes with mild steatosis, with small round vacuoles in the cytoplasm. The spleens of pMV262-JTY_0672-MS-infected mice had red and white medullas in the splenic tissue. The white medulla was large and irregularly shaped, with an occasional localized lax arrangement of cells around the central artery of the white medulla, and a reduction in the number of lymphocytes. The lungs of pMV262-JTY_0672-MS-infected mice had a small amount of granulocyte infiltration in the alveolar wall and eosinophilic material in a small number of alveoli. Macrophages were mostly seen oozing out of the alveoli, and a small number of leukocytes were seen in the blood vessels. The number of macrophages oozing out of the alveoli was increased when compared to the lungs of mice infected with the pMV262-MS strain. Analysis of the kidneys from pMV262-JTY_0672-MS- infected mice showed that the number of cells in the glomeruli was similar with the stroma. Hydropic degeneration of the renal tubular epithelial cells was common, with swollen cells and a sparsely stained cytoplasm. Eosinophilic material was seen in a small number of tubules with a small number of aggregates of cells with large nuclei and irregular shapes. Basophilic nuclei were evident in the mesangial stroma, surrounded by occasional lymphocytic infiltration (Figure 8F).

## Discussion

Previous research found that JTY_0672 was highly expressed in the BCG transcriptome following treatment with V_C_ (Song et al., 2020). Therefore, in this study, we constructed an pMV262-JTY_0672-BCG strain for primarily transcriptomic analysis. EMSA results showed that JTY_0672 could bind to the promoters of 43 target genes *in vitro*. Based on our EMSA results, the expression of 17 genes with known functions was verified by qRT-PCR, analysis showed that JTY_0672 is a broad-spectrum regulatory protein that can act as a repressor as well as an activator to regulate the transcription of genes. These findings are consistent with the characteristics of TetR family proteins reported previously (Ramos et al., 2005). Most of the target genes regulated by JTY_0672 can promote the growth of MS and the viability of macrophages. In dormancy, the latency antigen Rv1733c, as a membrane protein, is 12-fold upregulated in the overexpression transcriptome, and contains both T cell and B cell epitopes, which is a highly potential anti-tuberculosis vaccine candidate (Zhang et al., 2022). Rv3134c-Rv3133c (DosR) -Rv3132c (DosS) is an important operon in maintaining the dormant and resuscitation state of Mtb. These genes in this operon were all upregulated in the transcriptomic expression (Converse et al., 2009). PhoR is able to integrate nitrogen metabolism with hypoxia by the assistance of the hypoxia regulator DosR (Singh et al., 2020). These genes play an important role in promoting the growth and survival of Mtb and drug resistance, and our results found that JTY_0672 can directly regulate the expression of the above genes. Therefore, JTY_0672 represents a new molecular target for the treatment of TB.

TetR is one of the most abundantly expressed families of transcriptional regulatory proteins found in bacteria, and mainly act as a repressor (Ramos et al., 2005). For example, Rv3160c inhibits the expression of dioxygenase Rv3161c, and the *rv3160c-rv3161c* operon is involved in a variety of important physiological activities, including drug metabolism, lipid metabolism and cell wall synthesis (Tükenmez et al., 2021). The TetR regulatory protein AtsR is a redox agent that senses oxidative stress through thiol modification, thereby reducing antimicrobial resistance in bacteria (Su et al., 2022). However, a small number of TetR transcription factors can activate gene transcription. For example, DszGR can specifically activate the *dsz* operon, leading to enhanced biological desulfurization activity of bacteria (Keshav et al., 2022; Murarka et al., 2019).

*JTY_3148* (Rv3130c), also known as tgs1, is a diacylglycerol transferase and can accumulate triacylglycerol (TAG), which is closely related to the dormancy of Mtb (Sirakova et al., 2006). Mtb obtains fatty acids from the host for the synthesis of TAG, which is then stored in the form of intracellular lipid inclusion bodies to meet the carbon and nutrient requirements of bacteria during long-term retention. Nitrogen deficiency can promote the expression of *tgs1* and induce the accumulation of TAG (Santucci et al., 2019). In addition, tgs1 promotes the drug resistance of Mtb. Under the response of isoniazid, *tgs1* was found to be significantly up-regulated in MDR-Mtb, thus indicating that this gene could represent a significant breakthrough in the monitoring and treatment of drug resistant tuberculosis in the future (Yimcharoen et al., 2022). Given the important role of *rv3130c* in Mtb, it is extremely important to identify regulators that can modulate *rv3130c* expression. The binding of NarL-DevR, possibly as a heterodimer, can co-regulate the expression of the *rv3130c* promoter (Malhotra et al., 2015). Furthermore, Rv0348 has been shown to regulate genes involved in hypoxia (Rv3130c) (Abomoelak et al., 2009). In our study, transcriptomic analysis of JTY_0672 overexpression revealed high levels of *JTY_3148* expression. qRT-PCR confirmed that the expression of the *JTY_3148* gene increased by almost 16-fold, thus suggesting that JTY_0672 may act as an activator for *JTY_3148*. In addition, JTY_0672 specifically bound to the *JTY_3148* promoter both *in vitro* and *in vivo*, thus indicating that the JTY_0672 protein can directly regulate *JTY_3148* expression. Besides, JTY_0672 can enhance the resistance of MS to isoniazid.

Vitamins (Song et al., 2020; Schwalb et al., 2023), amino acids (Amalia et al., 2022; Medha et al., 2021), and metal ions (Sritharan, 2016) are known to play important roles in the infection, growth, and survival of Mtb. The TetR family of regulatory factors (TFRs) represent a large class of single component bacterial signal transduction systems, that can regulate the expression of key genes by recognizing environmental signals and trigger cellular responses to participate in physiological changes (Filipek et al., 2024). Although TFRs exhibit significant differences in terms of function and phylogenetic diversity, the proteins in the TFR are relatively similar in structure. The N-terminal region is a highly conserved sequence that can bind to DNA; however, the C-terminal region is more variable and can bind to ligands in its pocket, thus leading to protein multimerization and signal recognition (Yu et al., 2010). By EMSA, we found that V_B1_, V_B3_, V_B6_, V_C_, His, Cys, Asp, Glu, Fe^3+^, Pb^2+^, Cu^2+^, and Li^+^ were able to inhibit the binding of JTY_0672 to the *JTY_3148* promoter, thus suggesting that these cofactors may affect the function of transcription proteins. And growth tests show that cofactors such as V_C_, Fe^3+^, Cu^2+^ effect the growth of MS. We supposed that cofactors can inhibit the binding of JTY_0672 to the promoter region of targeted genes and reduce the bacteria growth by inhibiting the targeted genes expression (Supplementary Figure 5). Previous studies have reported that DosR can sense cofactors such as Arg, Lys, Glu, Fe^3+^, Cu^2+^, V_B6_, V_C_, and choline, thereby inhibiting protein and DNA binding (Cui et al., 2022). DnaK is known to inhibit the ATPase activity of DnaK by deforming its molecular structure, thus improving the efficacy of drug treatment and reducing rifampicin resistance (Hosfelt et al., 2022). Zn^2+^ affects the Fe-S system of Mtb via the interaction between sufS and sufU (Elchennawi et al., 2023). By studying TFR-cofactor interactions, it is important to identify the molecular mechanisms of bacteria, especially the mechanism responsible for multi-drug resistance.

The interaction between proteins and DNA is a very complex process. In the actual regulatory process in bacteria, both protein and DNA structures are three-dimensional; therefore, it is particularly important to analyze the three-dimensional structure of proteins. Typical proteins of the TetR family commonly possess nine conserved α-helices (Cuthbertson and Nodwell, 2013). In this study, the predicted 3D structure of JTY_0672 revealed 13 α-helices, this is similar to the structure of most of the other TetR proteins reported previously (Cuthbertson and Nodwell, 2013). The C-terminal structural domain of AcrR includes six helices, including helices α4 to α9. The large cavities generated by the C-terminal domain can form different drug binding sites (Routh et al., 2009). In our study, molecular docking revealed that the potential key amino acid residues of JTY_0672 that interact with cofactors include Leu-82, Arg-90, Thr-199, and Glu-220. Of these, both Leu-82 and Arg-90 in JTY_0672 are located in the C-terminal α6 helix, while Thr-199 and Glu-220 are located closer to the C-terminal α12 helix and α13 helix. The binding of ligands is associated with an increase in the degree of separation of the DNA binding domain of TFR. This will result in direct contact with the DNA binding domain, thus causing conformational changes in α4 and α6. Ligand binding causes α6 to undergo displacement, thus causing a pendulum movement in α4 (Cuthbertson and Nodwell, 2013). We hypothesize that this amino acid site located on the α6 helix is likely to be the key site for binding to cofactors, although point mutant proteins still need to be constructed to verify this hypothesis. In summary, the binding of cofactors may change the structure of the C-terminus of the protein, thereby affecting the affinity of JTY_0672 for DNA interaction, thereby influencing the expression levels of target genes.

In this study, we constructed an pMV262-JTY_0672-MS overexpression strain to investigate the physiological function of JTY_0672. Analysis showed that the overexpression of JTY_0672 significantly promoted the growth of MS and organ damage in experimental mice. As shown in Figure 6, CFU reduction was seen at later time points in the *in vitro* growth curve as well as in infection experiments. It is supposed that macrophages carried out the function of clearance to reduce the bacteria loads in the organs during infection process. This demonstrated that JTY_0672 plays a role in the pathogenicity of Ms, this finding is similar with the previous reported studies showing that TetR families are involved in Mtb growth and pathogenicity (Pandey et al., 2023). For instance, Δmce3R generated more intracellular ROS and demonstrated reduced susceptibility to oxidative stress, increased the frequency of generation of antibiotic persistence in Mtb, and promoted the colonization of Mtb in guinea pigs (Pandey et al., 2023).

Abnormal cytokine expression or receptor deficiency is an important cause of susceptibility to infectious diseases (Ritter et al., 2020). It was reported that modulation of cytokine-mediated immune responses in Mtb can inhibit its proliferation prevent harmful inflammatory immune responses (Cicchese et al., 2018; Domingo-Gonzalez et al., 2016; Erdmann et al., 2018). Antigen presenting cells are known to activate CD4 T cells upon receiving signaling molecules, which normally secrete interferon (IFN)γ and tumor necrosis factor (TNF), thus leading to the synergistic activation of anti-mycobacterial effector mechanisms and the secretion of pro-inflammatory cytokines (IL-6, IL-1β and TNF-α) in macrophages (Zeng et al., 2018).

IL-6 is considered a pro-inflammatory cytokine that activates the host immune response. However, IL-6 also has an inhibitory effect on macrophages. Mtb-infected macrophages secrete IL-6 and inhibit the immune response to IFN-γ in non-infected macrophages, thus suggesting that IL-6 also mediates anti-inflammatory mechanisms in TB (Rose-John, 2017). IFN-γ is a well known pro-inflammatory factor that plays an important role in the immune response against infection, and also participates in macrophage phagocytosis and cellular apoptosis in the host. IFN-γ stimulates the activity of immune cells to fight pathogens, including T cells and macrophages (Carabalí-Isajar et al., 2023; Januarie et al., 2022). TNF-α is known to be involved in the differentiation of T cells secreting Th1 cytokines, granuloma formation, and promotes the phagocytosis of Mφ and activation of epithelioid cells, ultimately leading to mycobacterial death (Carabalí-Isajar et al., 2023). IL-1β is crucial to establish and maintain T cell immunity against Th17-mediated Mtb (Diatlova et al., 2023; Lin et al., 2016). These cytokines can be used in the differential diagnosis of various stages of TB infection to assess the efficacy of treatment, and also represent new targets for the treatment of Mtb infection. In the present study, we found that JTY_0672 significantly promoted the colonization of MS in the lungs, liver, spleen, and kidneys of C57BL/6 mice, increased the serum levels of IL-6, IL-1β, INF-γ, and TNF-α, and facilitated inflammatory responses in infected mice. In this study, we found that JTY_0672 can promote MS infection and colonization, and affect the host inflammatory response. Other proteins in the TetR family have been reported previously to produce similar inflammatory responses (Gutiérrez et al., 2019). Collectively, our data demonstrate the physical functionality and regulatory role of JTY_0672 and enhance our understanding of the role of transcription regulators.

## Data Availability

The datasets presented in this study can be found in online repositories. The names of the repository/repositories and accession number(s) can be found in this article/Supplementary material.

## Footnotes

1
http://meme.nbcr.net/meme/

2
http://meme.nbcr.net/meme/cgi-bin/mast.cgi

## Supplementary material

Supplementary Table 1 Primers used in this study.

Supplementary Table 2 The gene expression level between pMV262-JTY_0672-BCG and pMV262-BCG by transcriptomics analysis.

Supplementary Table 3 Summary of potential targets for JTY_0672.
