# Supplementary material for: Novel target and cofactor repertoire for the transcriptional regulator JTY_0672 from Mycobacterium bovis BCG
Source: Front Microbiol. 2025 Jan 7;15:1464444. doi: 10.3389/fmicb.2024.1464444 (PMC11752888; doi:10.3389/fmicb.2024.1464444)
Supplement: Supplementary file 1 [file Data_Sheet_1.pdf]

Supplementary Material

1 Supplementary Figures and Tables

1.1 Supplementary Tables

Supplementary Table S1 Primers used in this study

Primer number	Primer sequences (5'-3')	Use (H37Rv homologous gene)
Cy5-SN494F	ATTCATGGTCAGCGCCTTCC	EMSA for *Cy5-JTY_3148* (*rv3130c*)
Cy5-SN494R	TCCAGACTCGTCGGGCATAC	EMSA for *Cy5-JTY_3148* (*rv3130c*)
Bio-SN494F	ATTCATGGTCAGCGCCTTCC	BLI for *Bio-JTY_3148* (*rv3130c*)
Bio-SN494R	TCCAGACTCGTCGGGCATAC	BLI for *Bio-JTY_3148* (*rv3130c*)
SN246F	ACACCCGAGACCGCCACACT	EMSA for *JTY_0001* (*rv0001*)
SN246R	CTTGGTTCCGGGTCGAGCTACAAC	EMSA for *JTY_0001* (*rv0001*)
SN261F	TCTATAGTCATCGATCCAGAGCCGCTTCG	EMSA for *JTY_0412* (*rv0404*)
SN261R	GCTGCGGTCCTTCGTCGGTCA	EMSA for *JTY_0412* (*rv0404*)
SN263F	GGTATTTGCAGCGGCGGCAGAGTC	EMSA for *JTY_1767* (*rv1753c*)
SN263R	TCAACTCGGGCACCGGGAATTTC	EMSA for *JTY_1767* (*rv1753c*)
SN266F	TCGGGCCCGTGATCGTCGAG	EMSA for *JTY_0673* (*rv0654*)
SN266R	CCGGGTTGGGGCCGTTACGC	EMSA for *JTY_0673* (*rv0654*)
SN441F	CTTTACCACCAGGGCACCAC	EMSA for *JTY_3151* (*rv3133c*)
SN441R	TGCTGCGGCACGCATTCGAG	EMSA for *JTY_3151* (*rv3133c*)
SN494F	ATTCATGGTCAGCGCCTTCC	EMSA for *JTY_3148* (*rv3130c*)
SN494R	TCCAGACTCGTCGGGCATAC	EMSA for *JTY_3148* (*rv3130c*)
SN496F	CATTTGATGCCTCCTAATCG	EMSA for *JTY_2045* (*rv2031c*)
SN496R	CGATCCTTGTCGAGGAACAG	EMSA for *JTY_2045* (*rv2031c*)
WH001F	TAATGAGGCTGGGAGCAACC	EMSA for *JTY_3929* (*rv3864*)
WH001R	ACGCTGGCGTTTTCAAGTTC	EMSA for *JTY_3929* (*rv3864*)
WH012F	AGGGTGTGTGTTGTCAGTGC	EMSA for *JTY_0720* (*rv0710*)
WH012R	ATCTAGCGTTGCGCCGAATA	EMSA for *JTY_0720* (*rv0710*)
WH025F	TGTGAATTGCTCGTGGCTCC	EMSA for *JTY_1747* (*rv1733c*)
WH025R	GCCGCAGTAACAGGATGAGG	EMSA for *JTY_1747* (*rv1733c*)
WH031F	CGACACGTAGTAAGCTGCCA	EMSA for *JTY_3919* (*rv3854c*)
WH031R	GATTCTCGGCAAGGGTCTGT	EMSA for *JTY_3919* (*rv3854c*)
SN300F	GCTCGAATACGTCTGCTACC	EMSA for *JTY_2023* (*rv2011c*)
SN300R	CGGCGAACTCTCGGAAAGAC	EMSA for *JTY_2023* (*rv2011c*)
SN448F	GCGCACTAGCCATCCCTGAC	EMSA for *JTY_0816* (*rv0793*)
SN448R	TGACCTTCTTCCGCTGCTAC	EMSA for *JTY_0816* (*rv0793*)
SN503F	CACGCCCAGAAGTTGACCAC	EMSA for *JTY_1752* (*rv1738*)
SN503R	ATGATGTTGCTCCCCTTTCCAGTG	EMSA for *JTY_1752* (*rv1738*)
SN504F	GAATGTTTGAGCCGCATGAG	EMSA for *JTY_2008* (*rv1996*)
SN504R	GCTGACATTGGCGTCCCCTCTG	EMSA for *JTY_2008* (*rv1996*)
SN505F	CATAGGTCACAAACGGCAAG	EMSA for *JTY_2019* (*rv2007c*)

SN505R	AACTGGCGAGGTTGGCAATC	EMSA for *JTY_2019* (*rv2007c*)
SN522F	GTGACGCAGTGGACGCATTG	EMSA for *JTY_3150* (*rv3132c*)
SN522R	ACGGTCGCTGCTGGACAATC	EMSA for *JTY_3150* (*rv3132c*)
SN498F	TTCGGCGTCGGAGGTTTGTG	EMSA for *JTY_1831* (*rv1813c*)
SN498R	GCGTGACAGGTGACGACTTG	EMSA for *JTY_1831* (*rv1813c*)
SN237F	GTAGGCGCCGAATAGTCGCGAGAC	EMSA for *JTY_1666* (*rv1652*)
SN237R	CGTTATGCAACAGTTTGCAAAC	EMSA for *JTY_1666* (*rv1652*)
SN255F	ACGCGGCTCAAACCGGAACG	EMSA for *JTY_0815* (*rv0792c*)
SN255R	GGGTAATCATTGCGTCCAAGAG	EMSA for *JTY_0815* (*rv0792c*)
SN298F	GTTCGTCAGGGTCGGTAGTC	EMSA for *JTY_3742* (*rv3682*)
SN298R	ATGCGTACTACAGTAGCGAC	EMSA for *JTY_3742* (*rv3682*)
SN423F	ACCTCGGGCATCCGGGCGAC	EMSA for *JTY_1164* (*rv1130*)
SN423R	TCCTGACCCGGCACATTTTG	EMSA for *JTY_1164* (*rv1130*)
SN424F	GCACAAGATTTCCACCGTCG	EMSA for *JTY_1165* (*rv1131*)
SN424R	CCCGGTCATCGAAAGATTCC	EMSA for *JTY_1165* (*rv1131*)
SN442F	TGACGGCGGATCGGAGATTG	EMSA for *JTY_3152* (*rv3134c*)
SN442R	GTCGATACCAACGACCACTG	EMSA for *JTY_3152* (*rv3134c*)
SN443F	CTGGAGCGGTTCGCAGTGTC	EMSA for *JTY_3254* (*rv3229c*)
SN443R	TCGCCATCGTCTTCTCCCTG	EMSA for *JTY_3254* (*rv3229c*)
SN445F	GGTGTGGTCCAGGGATATTC	EMSA for *JTY_3315* (*rv3290c*)
SN445R	CTATGATAGCAGGATTTACG	EMSA for *JTY_3315* (*rv3290c*)
SN451F	CGCCCTGATGGCTCCACAGC	EMSA for *JTY_0421* (*rv0412c*)
SN451R	CAACCGTCACGTCTGTTAGG	EMSA for *JTY_0421* (*rv0412c*)
SN452F	TAGCCGCCCATCTGTTCGAC	EMSA for *JTY_0696* (*rv0677c*)
SN452R	CTTCGGAACCAAAGAAAGTG	EMSA for *JTY_0696* (*rv0677c*)
SN454F	GACTACGAAGAGGCGTTGCG	EMSA for *JTY_0773* (*rv0752c*)
SN454R	TGACACGCTAACTCATTGTG	EMSA for *JTY_0773* (*rv0752c*)
SN455F	CCACAGCGGTCGTCGGATTG	EMSA for *JTY_0774* (*rv0753c*)
SN455R	AATCTGTGTGGTCATGGTTGTC	EMSA for *JTY_0774* (*rv0753c*)
SN456F	CAAGGCAACCTCCTCGGTTC	EMSA for *JTY_1042* (*rv1013*)
SN456R	ACGGCGATAGCTGCATCAAC	EMSA for *JTY_1042* (*rv1013*)
SN457F	AGATGGGCCAACTCTCGCAG	EMSA for *JTY_1607* (*rv1594*)
SN457R	ACAGTCATTGCCGCCCCTTC	EMSA for *JTY_1607* (*rv1594*)
SN458F	CTGGCTCGCTCGTCTTCATC	EMSA for *JTY_3074* (*rv3054c*)
SN458R	CGTTCCTTTCCGCTTGTTGG	EMSA for *JTY_3074* (*rv3054c*)
SN461F	GCCAACCAGCTTCCTCAGAC	EMSA for *JTY_3864* (*rv3800c*)
SN461R	TGGGTCCTCAGTCCGAAGTG	EMSA for *JTY_3864* (*rv3800c*)
SN465F	CAGCATGGGCAGCCAACAAG	EMSA for *JTY_3930* (*rv3865*)
SN465R	TAACTTTCTACTCGCTATGG	EMSA for *JTY_3930* (*rv3865*)
SN466F	AACGTAGAGGAGGGTTCAAC	EMSA for *JTY_3931* (*rv3866*)
SN466R	GATCAACCGAAAATCTTGTC	EMSA for *JTY_3931* (*rv3866*)
SN467F	CTAATTTCTCCGGTGATCTC	EMSA for *JTY_0529* (*rv0516c*)
SN467R	CAATAGCCTCGTTCTCCGTC	EMSA for *JTY_0529* (*rv0516c*)
SN469F	CACCAACCCTACCGCGACAC	EMSA for *JTY_1070* (*rv1040c*)
SN469R	TTGCTCCTTGTGTGTGAAAC	EMSA for *JTY_1070* (*rv1040c*)
SN470F	AGTTCGGGGTGTTGACGATG	EMSA for *JTY_1569* (*rv1542c*)
SN470R	AGCGTGACAGTAGTCCCATC	EMSA for *JTY_1569* (*rv1542c*)

SN475F	TGTTCCTCATCGCCGCATCG	EMSA for *JTY_2017* (*rv2005c*)
SN475R	GATGACAGCGTCGTGGTAGC	EMSA for *JTY_2017* (*rv2005c*)
SN476F	GGTTGATGCGGCTGGAAGAC	EMSA for *JTY_2044* (*rv2030c*)
SN476R	TCATCAGCACGGACCCAGTG	EMSA for *JTY_2044* (*rv2030c*)
SN478F	GATGAACCGGGCGACTCGAC	EMSA for *JTY_3865* (*rv3801c*)
SN478R	TCTCCTGTCACAAACATCTC	EMSA for *JTY_3865* (*rv3801c*)
SN479F	AGCAGAGCGGTGGTCAATTC	EMSA for *JTY_2009* (*rv1997*)
SN479R	ACCTCACCTCGCAGCCAGAC	EMSA for *JTY_2009* (*rv1997*)
Q11f	AGGTGGTGCGTCGTGGTCTG	qRT-PCR for *JTY_3151*
Q11r	GACAACAGATCGCGGCACAG	qRT-PCR for *JTY_3151*
Q12f	CTTATCGTCGCTCGCTCAAC	qRT-PCR for *JTY_3148*
Q12r	CGGCGATCAGCTCGAATAAC	qRT-PCR for *JTY_3148*
Q13f	GGTTGATGCGGCTGGAAGAC	qRT-PCR for *JTY_2045*
Q13r	TCGTCCTCGTCAGCACCTAC	qRT-PCR for *JTY_2045*
Q14f	GACGAACACGAAGGATTGAC	qRT-PCR for *JTY_1752*
Q14r	ACCTTCAACATTCGCTTCCC	qRT-PCR for *JTY_1752*
Q15f	GTCAGCCCAACAAACGAACC	qRT-PCR for *JTY_2008*
Q15r	GTTATTACCGGGGGCACGAC	qRT-PCR for *JTY_2008*
Q16f	CGGTAGTGAGTGCGTGGATG	qRT-PCR for *JTY_2019*
Q16r	GTAGATCGCCTTCCCAGTAG	qRT-PCR for *JTY_2019*
Q17f	CACGATAGCGCGTAGGGTTG	qRT-PCR for *JTY_3150*
Q17r	CAATGCGTCCACTGCGTCAC	qRT-PCR for *JTY_3150*
Q18f	GGTGTCCTGTGCTGCGTATG	qRT-PCR for *JTY_1164*
Q18r	CCACCTGGGCGATCTTGTAG	qRT-PCR for *JTY_1164*
Q19f	ACACCACCGCCATCTCCAAG	qRT-PCR for *JTY_1165*
Q19r	AACAACGCCAGCTCGGCATC	qRT-PCR for *JTY_1165*
Q20f	GGGCAGTGGTCGTTGGTATC	qRT-PCR for *JTY_3152*
Q20r	TGACGTACACCAGTCGCAGC	qRT-PCR for *JTY_3152*
Q22f	GTGACTTTGCCGTTCCGTTG	qRT-PCR for *JTY_2044*
Q22r	TGGTGTCAGCGCGGCTAAGC	qRT-PCR for *JTY_2044*
Q23f	GGACGATACTGCGGGTGTTC	qRT-PCR for *JTY_1747*
Q23r	TGGCTGCGGGAATCATGGAC	qRT-PCR for *JTY_1747*
Q24f	GCTTCTCCCTGACCGACAAC	qRT-PCR for *JTY_0673*
Q24r	GGACCGACTGGATCACCAAC	qRT-PCR for *JTY_0673*
Q25f	CATCGGGTAGCGGTCTTTGC	qRT-PCR for *JTY_3929*
Q25r	TTGTTTGAACAGCGACGAAC	qRT-PCR for *JTY_3929*
Q26f	GCGGCACCTGGGATTTGTTC	qRT-PCR for *JTY_3919*
Q26r	CAGGGACGGAATCGGAAACC	qRT-PCR for *JTY_3919*
Q27f	TCTCAAGCGTGCCTGGATAC	qRT-PCR for *JTY_0696*
Q27r	GGTCATCGGCGAAGACCTTC	qRT-PCR for *JTY_0696*
Q28f	CCTGTCACGCTTGGGATTCG	qRT-PCR for *JTY_3931*
Q28r	CCCAGGGTTTCGACCATCTC	qRT-PCR for *JTY_3931*
Bio-SNN535F	ATTCATGGTCAGCGCCTTCCCCGGTGATC CGGATTATCTGCAACCGTCAG	BLI for *JTY_3148*, 21bp, WT C-G at position 13
Bio-SNN535R	CTGACGGTTGCAGATAATCCGGATCACCG GGGAAGGCGCTGACCATGAAT	BLI for *JTY_3148*, 21bp, WT C-G at position 13
Bio-SNN536F	ATTCATGGTCAGCGCCTTCCCGGGTGATC CGGATTATCTGCAACCGTCAG	BLI for *JTY_3148*, 21bp, C-G change G-C at position 13

Bio-SNN536R	CTGACGGTTGCAGATAATCCGGATCACCCGGGAAGGCGCTGACCATGAAT	BLI for *JTY_3148*, 21bp, C-G change G-C at position 13
Bio-SNN537F	ATTCATGGTCAGCGCCTTCCCAGGTGATCCGGATTATCTGCAACCGTCAG	BLI for *JTY_3148*, 21bp, C-G change A-T at position 13
Bio-SNN537R	CTGACGGTTGCAGATAATCCGGATCACCTGGGAAGGCGCTGACCATGAAT	BLI for *JTY_3148*, 21bp, C-G change A-T at position 13
Bio-SNN538F	ATTCATGGTCAGCGCCTTCCCTGGTGATCCGGATTATCTGCAACCGTCAG	BLI for *JTY_3148*, 21bp, C-G change T-A at position 13
Bio-SNN538R	CTGACGGTTGCAGATAATCCGGATCACCAGGGAAGGCGCTGACCATGAAT	BLI for *JTY_3148*, 21bp, C-G change T-A at position 13
Q-SN494F	GAACCGTTCGCACGGTTTCG	qPCR for *JTY_3148* promotor
Q-SN494R	TTGAGGCAGGGCCAAAGGTC	qPCR for *JTY_3148* promotor
16S-SN494F	GAGATACTCGAGTGGCGAAC	16S rRNA
16S-SN494R	GGCCGGCTACCCGTCGTC	16S rRNA

Supplementary Table S3 Summary of potential targets for JTY_0672

Gene	Function	PCR size(bp)	EMSA
JTY_0001	chromosomal replication initiator protein DnaA	316	+
JTY_0412	long-chain-fatty-acid--AMP ligase FadD30	361	+
JTY_1767	PPE family protein PPE24	469	+
JTY_0673	carotenoid cleavage oxygenase	298	+
JTY_3151	two component transcriptional regulator DevR（DosR）	362	+
JTY_3148	diacyglycerol O-acyltransferase	309	+
JTY_2045	alpha-crystallin HspX	339	+
JTY_3929	ESX-1 secretion-associated protein EspE	332	+
JTY_0720	30S ribosomal protein S17	312	+
JTY_1747	transmembrane protein	301	+
JTY_3919	monooxygenase EthA	302	+
JTY_2023	hypothetical protein	438	+
JTY_0816	monooxygenase	300	+
JTY_1752	hypothetical protein	353	+
JTY_2008	universal stress protein	321	+
JTY_2019	Ferredoxin FdxA	329	+
JTY_3150	two component sensor histidine kinase DevS	348	+
JTY_1831	hypothetical protein	368	+
JTY_1666	N-acetyl-gamma-glutamyl-phoshate reductase argC	367	+
JTY_0815	transcriptional regulator	342	+
JTY_3742	bifunctional penicillin-insensitive transglycosylase/penicillin-sensitive transpeptidase ponA2	496	+
JTY_1164	2-methylcitrate dehydratase PrpD	381	+
JTY_1165	methylcitrate synthase PrpC	369	+
JTY_3152	universal stress protein	418	+
JTY_3254	stearoyl-CoA 9-desaturase desA3	378	+
JTY_3315	L-lysine-epsilon aminotransferase	355	+
JTY_0421	membrane protein	290	+

JTY_0696	membrane protein MmpS5	267	+
JTY_0773	acyl-CoA dehydrogenase FadE9	319	+
JTY_0774	methylmalonate-semialdehyde dehydrogenase	338	+
JTY_1042	polyketide synthase	307	+
JTY_1607	quinolinate synthetase A nadA	295	+
JTY_3074	hypothetical protein	343	+
JTY_3864	polyketide synthase	334	+
JTY_3930	ESX-1 secretion-associated protein EspF	258	+
JTY_3931	ESX-1 secretion-associated protein EspG	336	+
JTY_0529	anti-anti-sigma factor	320	+
JTY_1070	PE family protein PE8	316	+
JTY_1569	hemoglobin GlbN GlbN	322	+
JTY_2017	universal stress protein	299	+
JTY_2044	hypothetical protein	344	+
JTY_3865	long-chain-fatty-acid--AMP ligase FadD32	265	+
JTY_2009	cation transporter ATPase F ctpF	277	+

1.2 Supplementary Figures

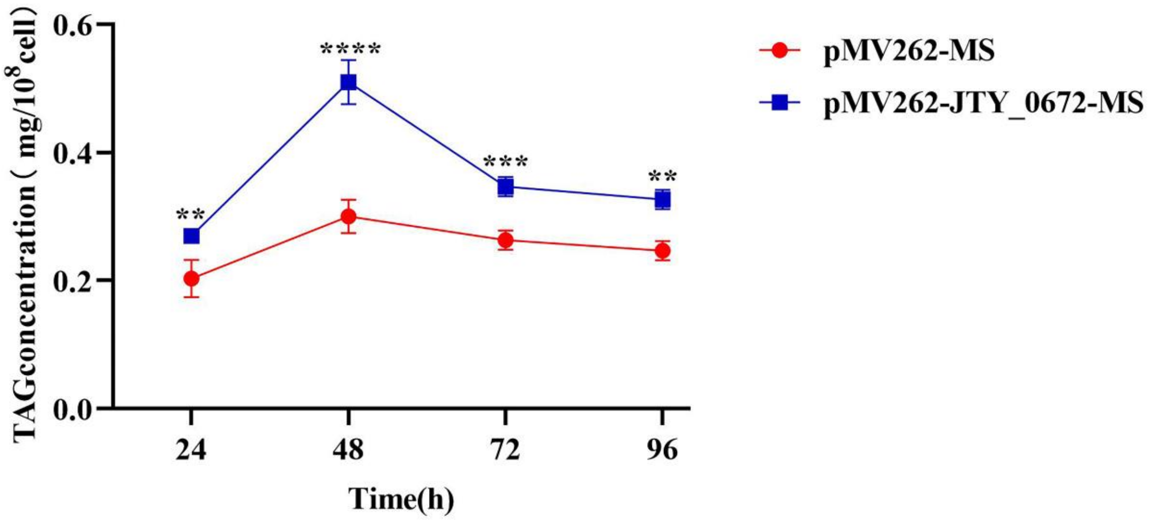

Supplementary Figure S1 Determination of TAG concentrations in the pMV262-MS and pMV262-JTY_0672-MS strains. The x-axis represents time, unit in hours, and the y-axis represents concentrations of TAG. Each sample was analyzed in triplicates. Error bars represent mean ± SEMs (n = 3), **, $p < 0.01$; ***, $p < 0.001$; ****, $p < 0.0001$.

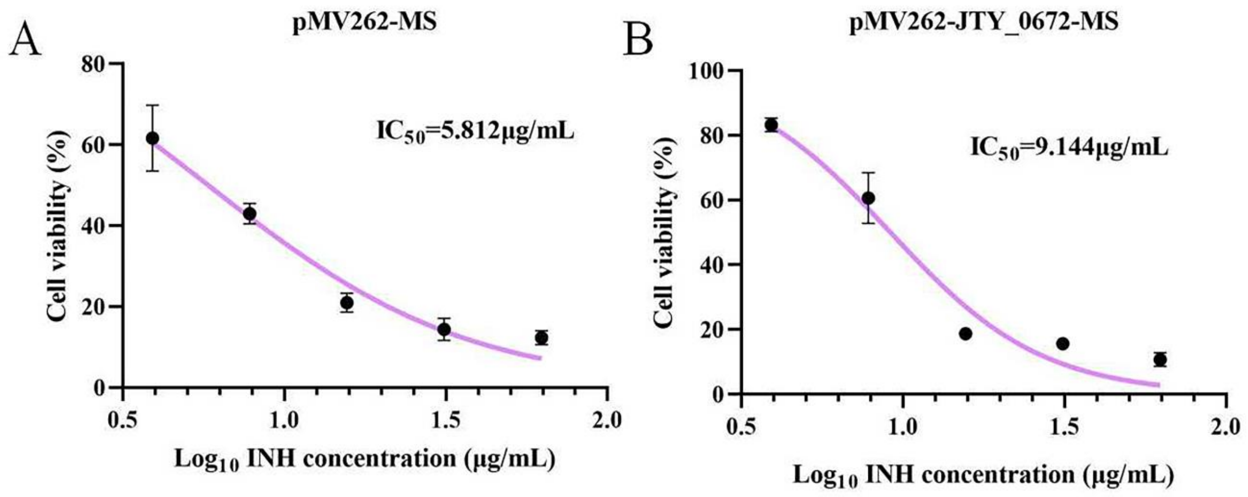

Supplementary Figure S2 Effect of isoniazid(INH) on pMV262-MS and pMV262-JTY_0672-MS proliferation. Cell viability of pMV262-MS (A) and pMV262-JTY_0672-MS (B) was determined by broth microdilution assay after treatment with INH. The x-axis represents concentrations of INH , and the Cell viability as the y-axis. Each sample was analyzed in triplicates. Error bars represent mean ± SEMs (n = 3).

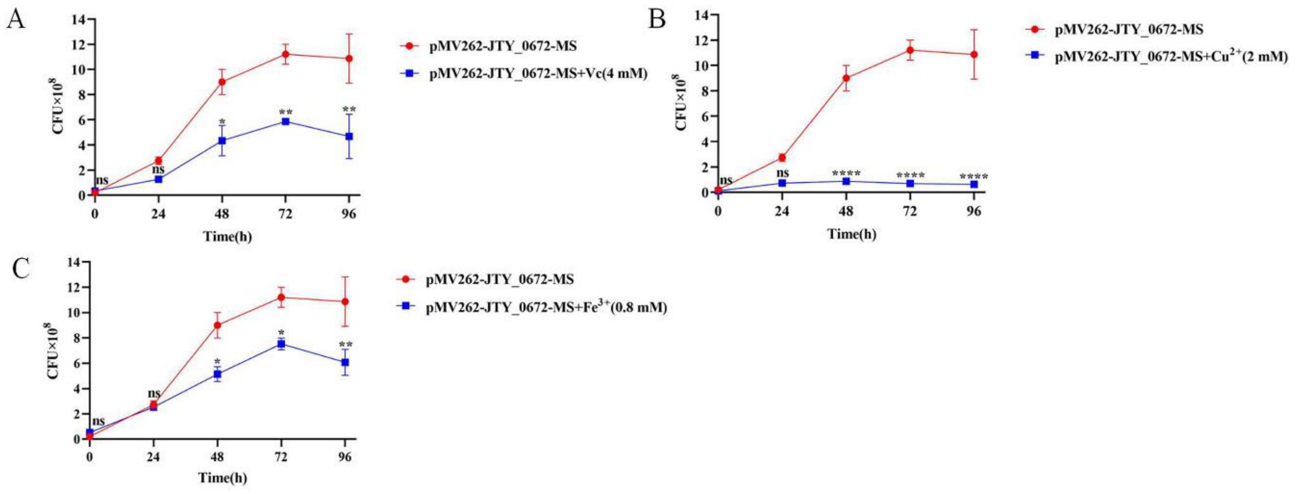

Supplementary Figure S3 Growth effect of different cofactors on the pMV262-JTY_0672-MS overexpressing strain. (A) V_C(4 mM). (B) Cu^{2+}(2 mM). (C) Fe^{3+}(0.8 mM). The growth curve was created using times as the x-axis and the number of bacteria as the y-axis. Each sample was analyzed in triplicates. Error bars represent mean ± SEMs (n = 3), ns stands for not significant, $p>0.05$; *, $p < 0.05$; **, $p < 0.01$; ****, $p < 0.0001$.

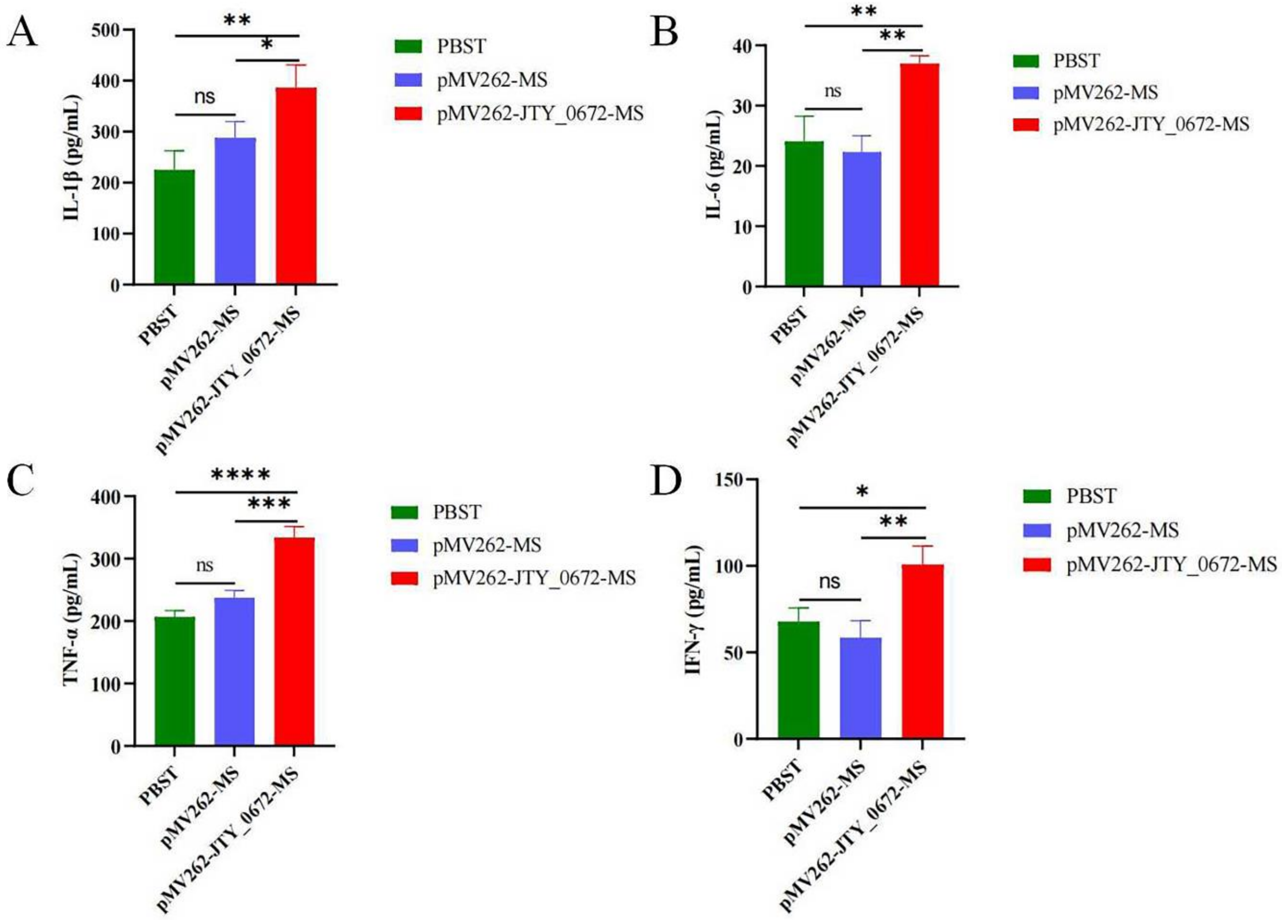

Supplementary Figure S4 Cytokine levels in the serum of infected 4d mice. (A) IL-1β. (B) IL-6. (C) TNF-α. (D) IFN-γ. The detection of cytokine levels from the immunized mice were performed using ELISA. Each sample was analyzed in triplicates. Error bars represent means ± SEMs (n = 3). Time is shown as the x-axis, and the number of cytokine levels as the y-axis. ns stands for not significant, $p > 0.05$; *, $p < 0.05$; **, $p < 0.01$; ***, $p < 0.001$; ****, $p < 0.0001$.

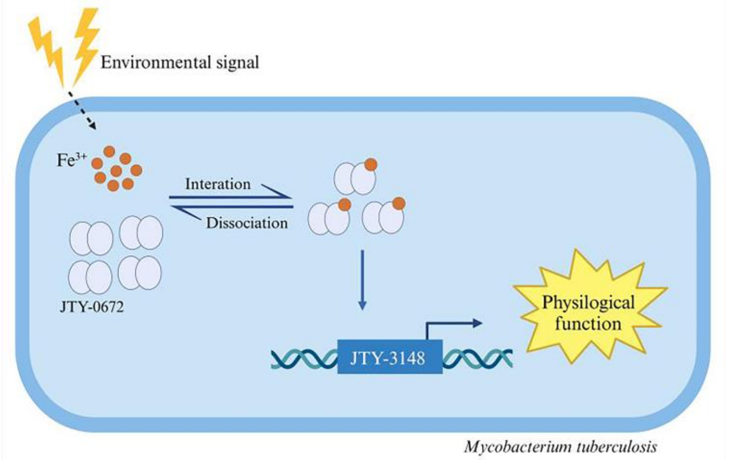

Supplementary Figure S5 A model showing that cofactor (Fe^{3+}) incorporate JTY_0672 to mediate bacterial physiological function in *Mycobacteria*. When the bacteria encounter the environmental signal stimulation, the level of Fe^{3+} changed rapidly. The concentration variation of Fe^{3+} mediated the binding affinity between JTY_0672 and the promoter *JTY_3148*, and affects the physiological function of Mtb by regulating the expression of genes.
